# Insecticide resistance profile of *Anopheles gambiae* from a phase II field station in Cové, southern Benin: implications for the evaluation of novel vector control products

**DOI:** 10.1186/s12936-015-0981-z

**Published:** 2015-11-18

**Authors:** Corine Ngufor, Raphael N’Guessan, Josias Fagbohoun, Krishanthi Subramaniam, Abibatou Odjo, Augustin Fongnikin, Martin Akogbeto, David Weetman, Mark Rowland

**Affiliations:** London School of Hygiene and Tropical Medicine, London, UK; Centre de Recherches Entomologiques de Cotonou, Cotonou, Benin; Pan African Malaria Vector Research Consortium, London, UK; Liverpool School of Tropical Medicine, Liverpool, UK

**Keywords:** Insecticide resistance, Experimental huts, Cové, Vector control product evaluation, *Anopheles gambiae* sl, Long-lasting insecticidal nets, Indoor residual spraying

## Abstract

**Background:**

Novel indoor residual spraying (IRS) and long-lasting insecticidal net (LLIN) products aimed at improving the control of pyrethroid-resistant malaria vectors have to be evaluated in Phase II semi-field experimental studies against highly pyrethroid-resistant mosquitoes. To better understand their performance it is necessary to fully characterize the species composition, resistance status and resistance mechanisms of the vector populations in the experimental hut sites.

**Methods:**

Bioassays were performed to assess phenotypic insecticide resistance in the malaria vector population at a newly constructed experimental hut site in Cové, a rice growing area in southern Benin, being used for WHOPES Phase II evaluation of newly developed LLIN and IRS products. The efficacy of standard WHOPES-approved pyrethroid LLIN and IRS products was also assessed in the experimental huts. Diagnostic genotyping techniques and microarray studies were performed to investigate the genetic basis of pyrethroid resistance in the Cové *Anopheles gambiae* population.

**Results:**

The vector population at the Cové experimental hut site consisted of a mixture of *Anopheles coluzzii* and *An. gambiae s.s.* with the latter occurring at lower frequencies (23 %) and only in samples collected in the dry season. There was a high prevalence of resistance to pyrethroids and DDT (>90 % bioassay survival) with pyrethroid resistance intensity reaching 200-fold compared to the laboratory susceptible *An. gambiae* Kisumu strain. Standard WHOPES-approved pyrethroid IRS and LLIN products were ineffective in the experimental huts against this vector population (8–29 % mortality). The L1014F allele frequency was 89 %. CYP6P3, a cytochrome P450 validated as an efficient metabolizer of pyrethroids, was over-expressed.

**Conclusion:**

Characterizing pyrethroid resistance at Phase II field sites is crucial to the accurate interpretation of the performance of novel vector control products. The strong levels of pyrethroid resistance at the Cové experimental hut station make it a suitable site for Phase II experimental hut evaluations of novel vector control products, which aim for improved efficacy against pyrethroid-resistant malaria vectors to WHOPES standards. The resistance genes identified can be used as markers for further studies investigating the resistance management potential of novel mixture LLIN and IRS products tested at the site.

**Electronic supplementary material:**

The online version of this article (doi:10.1186/s12936-015-0981-z) contains supplementary material, which is available to authorized users.

## Background

Malaria vector control and prevention today largely depends on the use of insecticides applied as indoor residual spray (IRS) or long-lasting insecticidal nets (LLINs). Both interventions are effective [1, 2] and have contributed immensely to reducing the burden of malaria in recent years [3]. Nevertheless, the rapid development and spread of insecticide resistance to the limited classes of insecticides approved for vector control poses a major threat to the gains so far achieved in malaria control [4]. The need for new insecticide products that can circumvent existing mechanisms of resistance to current insecticides has become critical.

There is currently concerted effort from the international vector control community towards the development of new public health insecticides. The present portfolio of the Innovative Vector Control Consortium (IVCC) for example, is designed to produce three entirely new classes of insecticides by 2023 with no cross-resistance to existing classes of insecticides [5]. The IVCC has set up several projects and some novel IRS and LLIN products have been taken to evaluation phase. The World Health Organization (WHO) through its pesticide evaluation scheme (WHOPES) has set criteria that such products must meet to obtain recommendation for large-scale use against malaria vectors. WHOPES guidelines require that these products be evaluated in Phase I laboratory, Phase II semi-field experimental hut studies and in Phase III randomized controlled trials in households or village clusters [6, 7].

Experimental huts are a good simulation of human-occupied houses which allow the performance of indoor vector control interventions/products to be measured in terms of their ability to kill mosquitoes, prevent feeding and deter mosquitoes from entering a home. Several experimental hut stations have been constructed across sub-Saharan Africa and used for evaluating various indoor vector control products/tools following WHOPES guidelines [8–13]. However, the resistance profile of the local vector populations in some of these studies was not fully characterized at the time of evaluation, making interpretation of results complex [14]. Considering the movement towards the evaluation of a new generation of vector control products that are expected to show improved efficacy against insecticide-resistant mosquitoes in order to attain WHO recommendation for large-scale use in pyrethroid resistant areas, it becomes imperative that the vector populations at experimental hut stations used for such evaluations are highly resistant and the profile of resistance and vector species is fully characterized and understood at the time of evaluation. In addition, because insecticide resistance tends to vary from one locality to another usually over very short distances, the local vector resistance profile reported for such Phase II evaluations has to be specific for the vicinity of the experimental hut station where the study was conducted. Furthermore, the new guidelines for substantiating efficacy claims of novel LLINs in areas of high resistance recently published by the Vector Control Advisory Group (VCAG) [15] specifies criteria that the vector populations in hut sites used for Phase II evaluations of such novel products should meet; the vector population should be well characterized and should have a pyrethroid resistance ratio of at least ten-fold compared to a susceptible laboratory strain.

The current study was designed to fully characterize insecticide resistance in the *Anopheles gambiae* sl vector population from an experimental hut station in Cové, Benin, a newly constructed site belonging to the Pan African Malaria Vector Research Consortium (PAMVERC) collaborative site between the London School of Hygiene and Tropical Medicine (LSHTM) and Centre de Recherches Entomologique de Cotonou (CREC) in parallel with a series of Phase II evaluations of novel IRS and LLIN products to WHOPES standards. Studies were also performed to assess year-round hut-entry rates in the Cové site. The efficacy of current WHOPES-approved pyrethroid IRS and LLINs was assessed in the experimental huts. The implications of the findings for the evaluation of the novel vector control tools aimed at improving the control of insecticide-resistant vector populations is discussed.

## Methods

### Cové site and experimental huts

**Fig. 1:**
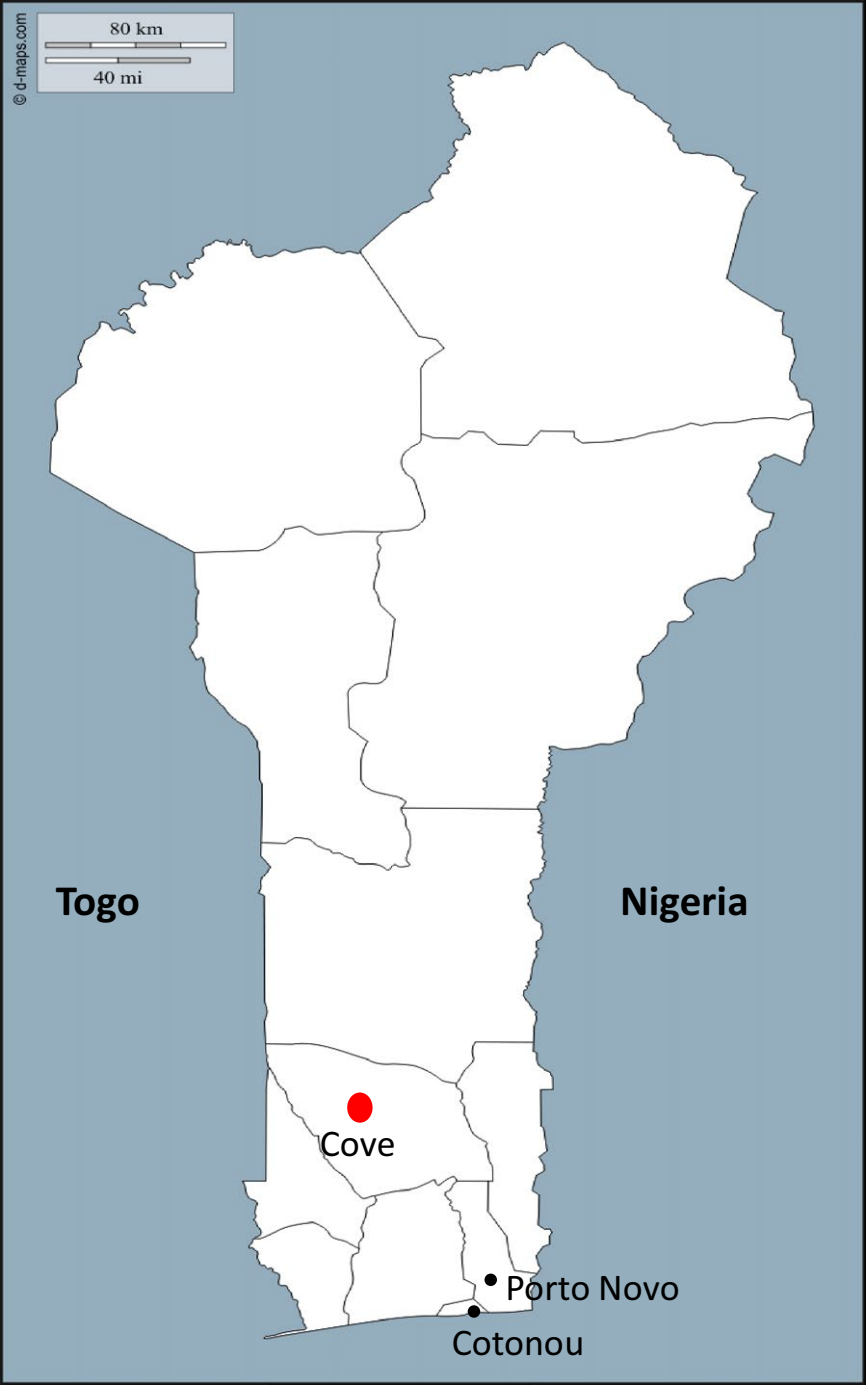
Map of Cové, Benin

The Cové Phase II experimental hut station is situated at the centre of a huge rice field in the city of Cové (7^o^14′N2^o^18′E) located 159 km from Cotonou, the economic capital of Benin (Fig. 1). The rice paddies provide extensive breeding sites for *An. gambiaes.l.* throughout the year. The rainy season extends from March to September and the dry season from October to February. Irrigated rice farming is done twice every year at the hut station, the first from March to May and the second from October to January. Seventeen experimental huts of the typical West African design described in WHOPES guidelines [6] have been constructed at this site by the PAMVERC collaborative unit between LSHTM and CREC.

To assess year-round variation in mosquito density and hut-entry rates in the experimental huts at Cové, consenting human volunteers slept under an untreated net in the huts and mosquitoes were collected daily over 12 months and brought to the laboratory for identification.

### WHO susceptibility bioassays

WHO susceptibility tests to assess the prevalence of resistance were performed using papers obtained from Universiti Sains Malaysia, impregnated with a range of insecticides from the four approved classes for vector control: namely, 0.75 % permethrin, 4 % DDT, 0.05 % deltamethrin, 0.1 % bendiocarb, 5 % malathion, and 4 % fenithrothion. Adult female mosquitoes 2–3 days old, which emerged from larvae collected from breeding sites next to the experimental huts, were exposed for 1 h to the insecticide-treated papers and mortality was recorded 24 h later. Approximately 100 mosquitoes (four replicates of 25 mosquitoes) were used per test and the average mortality was calculated. Control mosquitoes were exposed to untreated papers. Resistant females which remained alive after 24 h were kept for one more day until they were 3–4 days old, after which they were preserved submerged in RNA later for genotyping, target site resistance and microarray studies.

### Resistance intensity dose–response bioassay

To determine the intensity of resistance to pyrethroids in the wild *An. gambiae* Cové strain, mosquitoes that emerged from larvae collected from breeding sites at the experimental hut station were tested in CDC bottle bioassays treated with a range of doses of alphacypermethrin from 0.05 to 5 µg. Comparison was made with the laboratory-susceptible *An. gambiae* Kisumu strain tested with a dose range of 0.0005–0.05 µg. Alphacypermethrin was chosen for this study because it was one of the main pyrethroid insecticides used as a positive control in the parallel hut evaluations. A total of 100 mosquitoes were exposed for 1 h at each concentration and deaths were scored 24 h later. Log-dosage mortality curves were generated using probit analysis and estimates of the dose required to kill 50 % (LD50) of each strain and the resistance ratios relative to the susceptible laboratory strain were generated (PoloPlus version 1.0).

### Synergist bioassays

The effect of the insecticide synergist piperonyl butoxide (PBO), the primary action of which is to inhibit P450 mono-oxygenase enzymes, was evaluated in CDC bottle bioassays. One-hundred and fifty-two to five day old adult females were exposed (in batches of 25 mosquitoes) for 30 min in bottles treated with permethrin (21.5 µg/bottle) either alone or after 1 h pre-exposure to PBO (400 µg/bottle). Mosquitoes were also exposed to PBO alone. Control bottles were treated with acetone alone. The laboratory-susceptible Kisumu strain was also tested in bottles treated with permethrin alone for comparison. Mortality was recorded after 24 h.

### Efficacy of standard pyrethroid LLIN and IRS in experimental huts in Cové, Benin

To demonstrate the impact of pyrethroid resistance in Cové on the performance of standard WHOPES-approved pyrethroid LLINs and IRS products, hut data with pyrethroid treatments from four experimental hut trials that were performed in the Cové experimental huts station in parallel to this study were combined. These trials evaluated different novel IRS and LLIN products in comparison with the currently approved pyrethroid products following WHOPES guidelines. The results from the individual evaluation studies are being reported separately but for the current study, the mortality rates for mosquitoes entering huts treated with the WHOPES-approved pyrethroid LLINs and IRS were analysed and comparison made with an untreated control net. The following five treatments were included:

1. Untreated control net
2. Olyset Net (permethrin-incorporated LLIN)
3. Interceptor 1 (alphacypermethrin-coated LLIN)
4. Deltamethrin IRS applied at 25 mg/sq m
5. Alphacypermethrin IRS applied at 25 mg/sq m

During the trials, consenting human volunteers slept in the huts from dusk to dawn to attract mosquitoes. Mosquitoes were collected in the morning and brought to the laboratory for identification and scoring of mortality, blood feeding rate and exophily. The nets were rotated between huts to account for hut attractiveness to mosquitoes while the sleepers were rotated on successive nights to reduce any bias due to individual attractiveness to mosquitoes in accordance with a Latin square design.

### Ethical considerations

Chemoprophylaxis was provided to volunteer sleepers prior to the hut studies. Approval was obtained from the ethics review boards of the London School of Hygiene and Tropical Medicine and the Ministry of Health in Benin.

### Species identification and target site resistance

To identify vector species and target site resistance in Cové, DNA was extracted from a pair of legs taken from each female mosquito tested in the WHO susceptibility bioassays, and also from a set of unexposed mosquitoes (N = 89) collected from the Cové hut site at the beginning of the previous dry season (September–October 2014) for comparison between seasons. The legs were transferred to 96-well plates and extraction was done using a buffer-based boiling method. Species identification was conducted on each DNA sample using standard PCR techniques [16, 17]. TaqMan assays were used to characterize each sample for genotypes at three loci in the voltage-gated, sodium channel, target site (L1014F, L1015S and N1575Y) [18, 19] which confer resistance to DDT and pyrethroids, and also for the *ace*-*1* G119S resistance mutation in acetylcholinesterase, the target site of organophosphates and carbamates [20].

### Microarrays

**Fig. 2:**
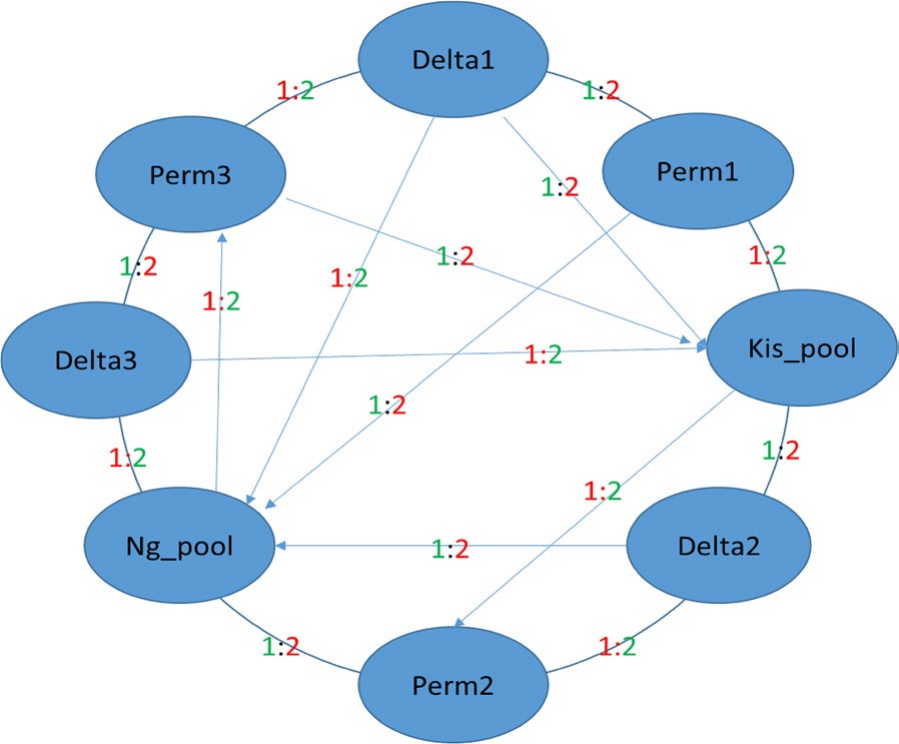
Interwoven loop design with three pools each of deltamethrin- (Delta) and permethrin-selected (Perm) samples from Cové, Benin, and one pool each from fully pyrethroid-susceptible colony samples (NGuosso and Kisumu). The outer loop connections run clockwise, directionality of inner connections is indicated by *arrows*: 1:2 indicates the dye label applied to the pool at the start and end of the connection, respectively. The R program MAANOVA [34] was used to analyse the interwoven loop data using previously described custom R-scripts [21]. No outlying quality microarrays were detected. Analysis used a threshold for gene discovery, which relied on consistency of a liberal *P* value threshold (P < 0.05), coupled with consistency in over-expression and a fold-change threshold of >2 across all deltamethrin and permethrin pools *vs* both susceptible pools

Total RNA was extracted from batches of ten mosquitoes that survived exposure to either deltamethrin or permethrin using the Ambion RNA extraction Kit. RNA quality and quantity was assessed using a NanoDrop spectrophotometer (Thermo Fisher Scientific) and a 2100 Bioanalyzer (Agilent Technologies) before further use. Three biological replicate extractions of total RNA from batches of ten mosquitoes for each treatment population were labelled and hybridized to the *An. gambiae* 15 k whole genome microarray using previously described protocols [21]. Expression profiles of females from pyrethroid-susceptible NGuosso and Kisumu strains, unexposed to insecticides, were compared to those of the permethrin and deltamethrin-selected Cové females using an interwoven loop design (Fig. 2). The design is optimized for two microarray slides (16 arrays) to maximize direct connections of primary interest (resistant *vs* susceptible).

## Results

### Monthly hut-entry rates

**Fig. 3:**
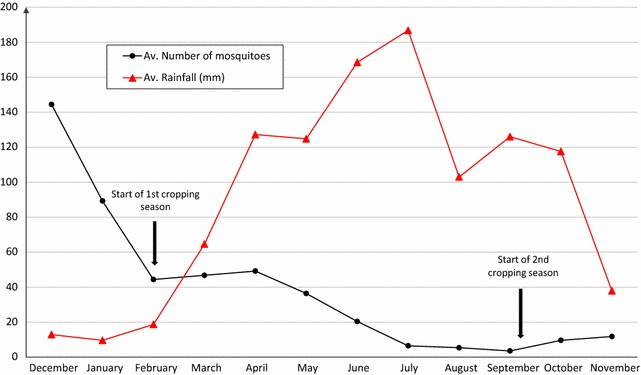
Monthly average night catch of *Anopheles gambiae* in untreated experimental huts in Cové, Benin

The average numbers of mosquitoes entering the experimental huts in Cové from December 2013 to November 2014 are presented in Fig. 3. The entry rates did not coincide with the rainy seasons; average numbers entering were highest during the drier months of the year (November to February) when rainfall is lowest in Cové. Peaks in monthly hut-entry rates occurred 2 months into the first and second rice planting (49/hut/night in April and 144/hut/night in December, respectively) thus showing synchrony with rice planting seasons in Cové. The lowest entry rate was recorded between July and September (3.5–6.5/hut/night).

### WHO susceptibility bioassays

**Fig. 4:**
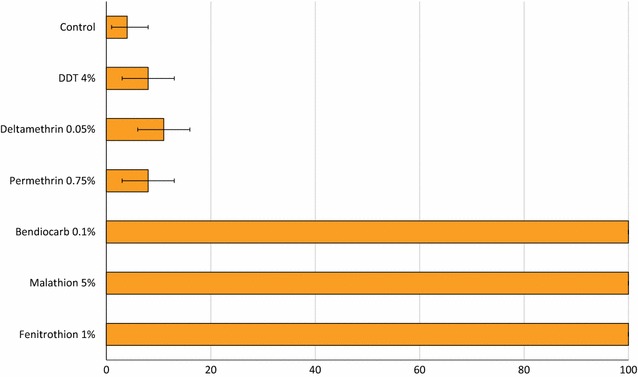
Mortality (%) of wild *Anopheles gambiae* sl from the experimental hut station Cové, Benin exposed to diagnostic doses of insecticides in WHO cylinder bioassays. *Error bars* represent 95 % confidence intervals

The mortality rates of the wild Cové strain exposed to diagnostic doses of insecticides in WHO susceptibility bioassays are presented in Fig. 4. Mortality in the control was 4 %. Mortality with the organochlorine DDT and with pyrethroids was less than 15 % indicating a high prevalence of resistance to both classes of insecticide. Bendiocarb, malathion and fenitrothion induced 100 % mortality demonstrating full susceptibility to organophosphates and carbamates.

### Resistance intensity bioassays

The results on dose–response studies comparing the Cové and Kisumu strain are presented in Table 1. The LD50 values were 0.0004 µg/ml for Kisumu and 0.083 µg/ml for the Cové strain. This resulted in a resistance ratio (RR) of 200-fold in the wild Cové compared to the Kisumu strain.

**Table 1:** Intensity of pyrethroid (alpha-cypermethrin) resistance in *Anopheles gambiae* sl from experimental hut station in Cové, Benin

Strain	Slope (SE)	LD50 (95 % CI)	LD95 (95 % CI)	RR 50 (95 % CI)
Kisumu	1.0 (0.15)	0.0004 (0.0–0.01)	0.01 (0.002–3.64)	–
Cové	1.3 (0.35)	0.083 (0.04–0.1)	1.49 (0.8–4.4)	200 (120.3–315.8)

Lethal doses (LD) are expressed in µg/ml

### PBO synergist bioassays

**Fig. 5:**
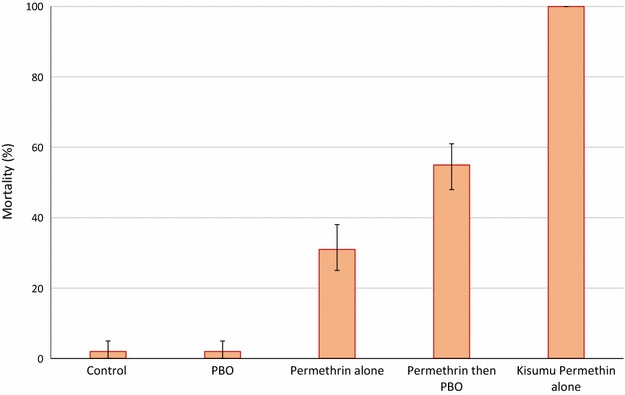
Mortality (%) of wild *Anopheles gambiae* sl from Phase II experimental hut station Cové, Benin exposed to permethrin in CDC bottle bioassays with and without the synergist PBO. *Error bars* represent 95 % confidence intervals

Mortality in CDC bottles treated with permethrin alone was 100 % with the Kisumu strain but only 32 % with the Cové population (Fig. 5). Pre-exposure of the Cové population to PBO resulted in a significant increase in mortality to 50 % (P < 0.05), suggesting at least some involvement of P450 detoxification enzymes in permethrin resistance in this vector population.

### Efficacy of standard pyrethroid LLIN and IRS in experimental huts in Cové, Benin

**Fig. 6:**
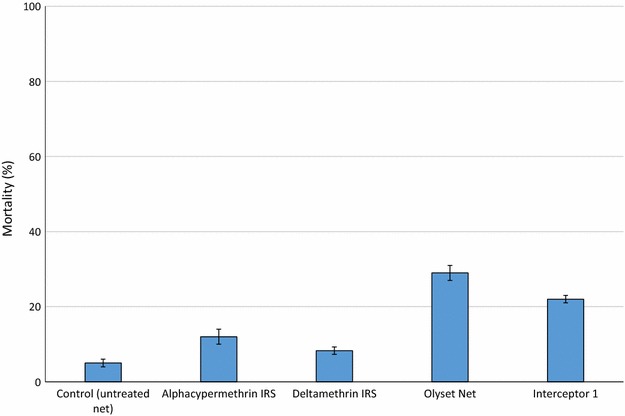
Mortality (%) of pyrethroid resistance *Anopheles gambiae* sl in experimental huts in Cové, Benin treated with standard pyrethroid LLINs and IRS products. *Error bars* represent 95 % confidence intervals

Results of the efficacy of standard pyrethroid LLINs and IRS in experimental huts in Cové, Benin are presented in Fig. 6. Mortality with the control net was 5 %. Mortality did not exceed 30 % with any of the pyrethroid treatments tested. The pyrethroid LLINs induced higher mortality rates (29 % with Olyset Net and 22 % with Interceptor 1) than the pyrethroid IRS treatments (8 % with deltamethrin and 12 % with alphacypermethrin).

### Species identification

Species identification PCR results revealed that the vector population was 100 % *An. gambiae* (N = 187). SINE PCR results showed that the population consisted of a mixture of the *Anopheles coluzzii* and *An. gambiaes.s*., the proportions of which showed seasonal variation. Twenty-three per cent of 89 samples collected in the dry season were *An. gambiaes.s.* while no *An. gambiaes.s.* was found in samples collected during the rainy season (N = 169); Chi-sq = 29.2, 1 df, P = 6 × 10^−8^.

### Target site resistance alleles

Overall, 1014F allele frequency in the Cové population was 89 % (n = 247), and this did not vary significantly between samples collected in the rainy and dry seasons (P > 0.05) (Table 2). No 1014S alleles were found, and only one heterozygote for the N1575Y mutation was identified. The *ace*-*1* 119S allele was also very rare.

**Table 2:** Frequency of target site resistance alleles in *Anopheles gambiae* sl from experimental hut station in Cové, Benin

Resistance gene	RR	RS	SS	Total tested	Resistant allele freq (%)
L1014F	200	41	6	247	89
*Ace*-*1* 119S	0	2	178	180	0.6
N1575Y	0	1	177	178	0.3

*RR* homozygous resistant, *RS* heterozygous, *SS* homozygous susceptible

### Microarray results

**Fig. 7:**
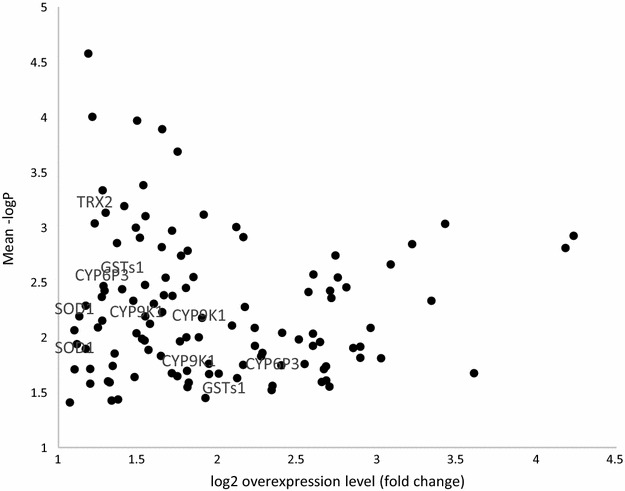
Genes significantly over-expressed (relative to susceptibles) in pyrethroid-resistant *Anopheles gambiae* sl from experimental hut station in Cové, Benin

Genes meeting the thresholds for significant over-expression in the microarray studies are presented in Fig. 7. A total of 113 probes covering 83 genes were significantly over-expressed in the Cové deltamethrin- and permethrin-resistant pools relative to the Kisumu and NGousso pools, and several of these are known detoxification genes. Using equivalent criteria for under-expression, 16 probes covering 12 genes were detected. The majority of the significantly differentially expressed genes do not have known roles in insecticide resistance, including those with highest statistical confidence and fold-change (the full table of results is given in Additional file 1). Of the detoxification genes, highlighted in Fig. 7, there are two cytochrome P450s, CYP6P3 and CYP9K1, a glutathione S-transferase GSTs1, a thioredoxin gene, TRX2, and superoxide-dismutase, SOD1. The best known of these genes in terms of resistance association, CYP6P3, is over-expressed in Cové at an average level of approximately four times compared to the susceptible strains.

## Discussion

The current study was designed to provide an in-depth characterization of the genotype and resistance profile of *An. gambiaes.l.* at a newly constructed experimental hut station in Cové, Benin, being used for WHOPES Phase II evaluation of novel IRS and LLIN products that are expected to show improved performance against pyrethroid-resistant mosquito vectors and potentially manage resistance. Characterizing the vector population in an experimental hut site is crucial to the accurate interpretation of results obtained in the evaluation of such products.

For any given experimental hut study, a minimum average hut-entry rate is usually required for the study to have enough statistical power to demonstrate the expected impact [22]. As expected of most rice growing areas in West Africa, the Cové experimental hut site is characterized by very high mosquito vector densities, which resulted in high hut-entry rates, making the site suitable for most Phase II product evaluation studies. Nevertheless, the results also showed seasonality in vector density linked to the rice cropping season with the lowest hut entry recorded in the months of June–September, a period during which no rice growing activity is ongoing and rice paddies are dry. Assuming that the rice cropping seasons in Cové remain unchanged, plans have been made to exclude this period where possible when planning hut evaluation studies in this site.

The resistance bioassays demonstrated very high levels of phenotypic resistance to pyrethroids and DDT in the Cové wild *An. gambiaes.l.* population, revealing a resistance ratio of 20-fold (compared to a susceptible laboratory strain) which far exceeds the minimum of ten-fold set by the VCAG for the evaluation of new LLINs with efficacy claims against highly pyrethroid-resistant mosquitoes [15]. This was confirmed by the low mortality rates (<30 %) achieved with standard WHOPES-approved pyrethroid LLINs and IRS in the experimental huts in Cové. Mortality rates of >80 % with pyrethroid IRS and insecticide-treated nets have been reported in earlier experimental hut studies in a previously pyrethroid-susceptible area in Benin [23]. The findings here constitute further evidence of the poor performance of current pyrethroid IRS and LLIN products when confronted with highly pyrethroid-resistant vector populations [9, 14, 23, 24]. The novel IRS and LLIN products being tested in the experimental huts in Cové involve non-pyrethroid classes of insecticide with new modes of action to which there are currently no records of resistance in malaria vectors; hence, improved mortality rates are expected.

The genotyping results revealed that the vector population in the Cové experimental hut station is composed of a mixture of *An. coluzzii* and *An. gambiaes.s.* with their relative abundance varying through the course of the year. While all samples genotyped from the March cropping season (start of the rainy season in 2014) were *An. coluzzi*, *An. gambiaes.s.* was found in a smaller proportion (23 %) only in samples that were collected at the start of the October annual rice cropping season in the Cové site (start of the dry season in 2013). Seasonal variation in these two sub-species of *An. gambiae* has been reported in several areas in West Africa [25, 26]. Unlike the *An. coluzzi* which is well adapted to rice paddies in West Africa, *An. gambiaes.s.* tends to occupy transitory man-made breeding sites, such as pits, ponds and puddles which are more likely to exist in the dry season in Cové. Nevertheless, because both species contribute similarly to disease transmission and are anthropophilic and endophilic [26], they can be equally targeted by the various novel IRS and LLIN products being evaluated in the experimental huts in Cové. Further studies are underway to investigate the extent of the variation in both vector sub-species in Cové through the course of the year, in relation to other human activities at the site and to assess the impact it may have on phenotypic resistance to pyrethroids.

The resistance genotyping studies suggests that the pyrethroid resistance encountered in the Cové vector population could be partly attributable to high frequencies of the 1014F kdr allele (0.89 allele frequency), with the 1014S mutation absent and the 1575Y allele very rare. The latter should be monitored however because it can effectively double the resistance conferred by 1014F alone [19]. *Ace*-*1* 119S was also very rare, which likely explains the lack of any carbamate or organophosphate resistance. However, again this requires monitoring because it is strongly diagnostic of resistance to each insecticide class [27] and can confer very high resistance in combination with P450 metabolic enzymes [28]. Indeed, some vector populations in West Africa with high frequencies of pyrethroid resistance mutations and the *ace*-*1* 119S mutation are resistant to all current classes of insecticides [29]. The PBO synergism data suggested a moderate impact of P450 enzymes in resistance, which was confirmed by the microarray study that identified two P450 genes as over-expressed in pyrethroid-selected *An. coluzzi* in Cové. Of these, CYP6P3 has been previously validated as a metabolizer of class I and II pyrethroids [30] and bendiocarb to a lesser extent [28]. CYP6P3 has been reported as over-expressed in pyrethroid-resistant field populations of *An. gambiaes.l.* from Dodowa in Ghana [31], Akron and Gbejromede in Benin, Orugun in Nigeria [32], and Tiassale in Côte d’Ivoire [28] and is thus operationally considered a key diagnostic marker of pyrethroid resistance. Further studies will be needed to investigate the other genes which were over-expressed in the microarray study for their roles in conferring pyrethroid resistance.

## Conclusion

In addition to improving the control of pyrethroid-resistant malaria vectors, the novel LLIN and IRS products evaluated at the Cové site are expected to also manage pyrethroid resistance since they are treated with insecticide mixtures [4, 33]. To investigate this, surviving mosquitoes from the different experimental hut treatments have been preserved in RNAlater for further studies comparing their capacity to prevent selection on insecticide resistance genes. Based on the microarray results obtained in the present study, CYP6P3 can be considered a suitable candidate gene for investigating selection of metabolic resistance to pyrethroids in follow-up quantitative real time PCR studies.

## Additional file

10.1186/s12936-015-0981-z Full microarray results.

## References

[1] Lengeler C: Insecticide treated bed nets and curtains for preventing malaria. Cochrane Database Syst Rev 2004;CD000363. 10.1002/14651858.CD000363.pub2 15106149

[2] Pluess B, Tanser FC, Lengeler C, Sharp BL. Indoor residual spraying for preventing malaria. Cochrane Database Syst Rev 2010; CD006657. 10.1002/14651858.CD006657.pub2 PMC6532743 20393950

[3] WHO. World malaria report 2012. Geneva: World Health Organization, 2012.

[4] WHO: Global plan for insecticide resistance management. Geneva: World Health Organization, 2012.

[5] Hemingway J (2014). The role of vector control in stopping the transmission of malaria: threats and opportunities. Philos Trans R Soc.

[6] WHO: Guidelines for laboratory and field testing of long‐lasting insecticidal nets. World Health Organisation 2013:WHO/HTM/NTD/WHOPES/2013.2013.

[7] WHO. Guidelines for testing mosquito adulticides for indoor residual spraying (IRS) and for treatment of mosquito nets (ITNs). WHO/CDS/WHOPES/GCDPP/20063, Geneva:World Health Organization, 2006.

[8] Koudou BG, Koffi AA, Malone D, Hemingway J (2011). Efficacy of PermaNet^®^ 2.0 and PermaNet^®^ 3.0 against insecticide-resistant* Anopheles gambiae* in experimental huts in Côte d’Ivoire. Malar J.

[9] Ngufor C, Chouaïbou M, Tchicaya E, Loukou B, Kesse N, N’Guessan R (2014). Combining organophosphate-treated wall linings and long-lasting insecticidal nets fails to provide additional control over long-lasting insecticidal nets alone against multiple insecticide-resistant* Anopheles gambiae* in Côte d’Ivoire: an experimental hut trial. Malar J.

[10] Ngufor C, N’guessan R, Fagbohoun J, Odjo A, Malone D, Akogbeto M (2014). Olyset Duo^®^ (a pyriproxyfen and permethrin mixture net): an experimental hut trial against pyrethroid resistant* Anopheles gambiae* and Culex quinquefasciatus in Southern Benin. PLoS One.

[11] Ngufor C, Tungu P, Malima R, Kirby M, Kisinza W, Rowland M (2014). Insecticide-treated net wall hangings for malaria vector control: an experimental hut study in north-eastern Tanzania. Malar J.

[12] Tungu P, Magesa S, Maxwell C, Malima R, Masue D, Sudi W (2010). Evaluation of PermaNet 3.0 a deltamethrin-PBO combination net against* Anopheles gambiae* and pyrethroid resistant Culex quinquefasciatus mosquitoes: an experimental hut trial in Tanzania. Malar J.

[13] Pennetier C, Bouraima A, Chandre F, Piameu M, Etang J, Rossignol M (2013). Efficacy of Olyset Plus, a new long-lasting insecticidal net Incorporating permethrin and piperonil-butoxide against multi-resistant malaria vectors. PLoS One.

[14] Strode C, Donegan S, Garner P, Enayati AA, Hemingway J (2014). The impact of pyrethroid resistance on the efficacy of insecticide-treated bed nets against African anopheline mosquitoes: systematic review and meta-analysis. PLoS Med.

[15] WHO: Third meeting of the Vector Control Advisory Group. Geneva. World Health Organization, 2015.

[16] Scott JA, Brogdon WG, Collins FH (1993). Identification of single specimens of the* Anopheles gambiae* complex by the polymerase chain reaction. Am J Trop Med Hyg.

[17] Fanello C, Santolamazza F, della Torre A (2002). Simultaneous identification of species and molecular forms of the* Anopheles gambiae* complex by PCR-RFLP. Med Vet Entomol.

[18] Bass C, Nikou D, Donnelly MJ, Williamson MS, Ranson H, Ball A (2007). Detection of knockdown resistance (kdr) mutations in* Anopheles gambiae*: a comparison of two new high-throughput assays with existing methods. Malar J.

[19] Jones CM, Liyanapathirana M, Agossa FR, Weetman D, Ranson H, Donnelly MJ (2012). Footprints of positive selection associated with a mutation (N1575Y) in the voltage-gated sodium channel of* Anopheles gambiae*. Proc Nat Acad Sci USA.

[20] Bass C, Nikou D, Vontas J, Donnelly MJ, Williamson MS, Field LM (2010). The Vector Population Monitoring Tool (VPMT): high-throughput DNA-based diagnostics for the monitoring of mosquito vector populations. Malar Res Treat.

[21] Mitchell SN, Stevenson BJ, Muller P, Wilding CS, Egyir-Yawson A, Field SG (2012). Identification and validation of a gene causing cross-resistance between insecticide classes in* Anopheles gambiae* from Ghana. Proc Nat Acad Sci USA.

[22] Johnson PC, Barry SJ, Ferguson HM, Müller P (2015). Power analysis for generalized linear mixed models in ecology and evolution. Methods Ecol Evol.

[23] N’Guessan R, Corbel V, Akogbeto M, Rowland M (2007). Reduced efficacy of insecticide-treated nets and indoor residual spraying for malaria control in pyrethroid resistance area, Benin. Emerg Infect Dis.

[24] Asidi A, N’Guessan R, Akogbeto M, Curtis C, Rowland M (2012). Loss of household protection from use of insecticide-treated nets against pyrethroid-resistant mosquitoes, Benin. Emerg Infect Dis.

[25] Dabiré KR, Diabaté A, Djogbenou L, Ouari A, N’Guessan R, Ouédraogo J-B (2008). Dynamics of multiple insecticide resistance in the malaria vector* Anopheles gambiae* in a rice growing area in South-Western Burkina Faso. Malar J.

[26] Lehmann T, Diabate A (2008). The molecular forms of* Anopheles gambiae*: a phenotypic perspective. Infect Genet Evol.

[27] Essandoh J, Yawson AE, Weetman D (2013). Acetylcholinesterase (Ace-1) target site mutation 119S is strongly diagnostic of carbamate and organophosphate resistance in* Anopheles gambiae* s.s. and Anopheles coluzzii across southern Ghana. Malar J.

[28] Edi CV, Djogbénou L, Jenkins AM, Regna K, Muskavitch MA, Poupardin R (2014). CYP6 P450 enzymes and ACE-1 duplication produce extreme and multiple insecticide resistance in the malaria mosquito* Anopheles gambiae*. PLoS Genet.

[29] Edi C, Koudou BG, Jones CM, Weetman D, Ranson H (2012). Multiple-insecticide resistance in* Anopheles gambiae* mosquitoes, Southern Côte d’Ivoire. Emerg Infect Dis.

[30] David JP, Ismail HM, Chandor-Proust A, Paine MJ (2013). Role of cytochrome P450s in insecticide resistance: impact on the control of mosquito-borne diseases and use of insecticides on Earth. Philos Trans R Soc Lond B Biol Sci.

[31] Müller P, Warr E, Stevenson BJ, Pignatelli PM, Morgan JC, Steven A (2008). Field-caught permethrinresistant* Anopheles gambiae* overexpress CYP6P3, a P450 that metabolises pyrethroids. PLoS Genet.

[32] Djouaka RF, Bakare AA, Coulibaly ON, Akogbeto MC, Ranson H, Hemingway J, Strode C (2008). Expression of the cytochrome P450s, CYP6P3 and CYP6M2 are significantly elevated in multiple pyrethroid resistant populations of* Anopheles gambiae* s.s. from Southern Benin and Nigeria. BMC Genom.

[33] Denholm I, Rowland MW (1992). Tactics for managing pesticide resistance in arthropods: theory and practice. Annu Rev Entomol.

[34] Wu H, Kerr K, Cui X, Churchill G: MAANOVA: a software package for the analysis of spotted cDNA microarray experiments. In: The analysis of gene expression data: methods and software. New York: Springer, 2002. p. 313–341.

